# Historical Review and Clinical Uses of Skin Indentation to Assess Limb Lymphedema

**DOI:** 10.7759/cureus.79829

**Published:** 2025-02-28

**Authors:** Harvey N Mayrovitz

**Affiliations:** 1 Medical Education, Nova Southeastern University Dr. Kiran C. Patel College of Allopathic Medicine, Davie, USA

**Keywords:** bcrl, breast cancer, gynecological cancer, indentation resistance, lymphedema, lymphedema measurement, lymphedema treatment, prostate cancer, tonometry

## Abstract

Limb lymphedema occurs when the drainage capacity of the limb's lymphatic system is less than needed to remove fluid filtered from the capillary network. This happens due to reduced lymphatic network function or increased resistance to lymph flow within the network. Upper extremity lymphedema may result from breast cancer treatment, and lower extremity lymphedema may occur after treatments for gynecological or prostate cancer. There are multiple other causes of limb lymphedema, with filariasis being the greatest cause worldwide. Over time, lymphedema causes changes in the skin and underlying tissue, which affect the tissue’s mechanical properties. Consequently, measurements of changes in the skin's compressibility may help evaluate the magnitude of progressive increases in lymphedema or help evaluate treatment-related improvements. A noninvasive parameter to assess such changes is the skin’s tissue indentation resistance (TIR), expressed as the tissue indentation force (TIF) divided by the tissue indentation distance (TID). The TIR increases as the tissue becomes more fibrotic and decreases with effective treatment. This review aims to describe the various indentation methods and their applications and report on the clinical findings and utility of such measurements related to limb lymphedema due to any cause. Fortuitously, the author has direct experimental experience in this area. Three databases (Web of Science, PubMed, and Embase) were searched for peer-reviewed, original study articles on skin indention in limb lymphedema written in English. After screening the initial 236 articles, 50 studies were retrieved and evaluated for relevancy. Of these, 27 met the inclusion criteria and were relevant. In 18 studies, indentation was used to evaluate lymphedema treatment, with 11 using a fixed TIF and 7 using a fixed TID device. The remaining nine studies used indentation to characterize aspects of established limb lymphedema were evaluated. The present review's findings and the images provided clarify the evolution of tissue indentometry as an adjunctive tool in assessing limb lymphedema over the past 38 years. Its uses range from evaluating naturally occurring changes in lymphedematous skin using shallow and deep indentations to using these measures to evaluate various lymphedema treatment modalities. In all cases investigated, whether indentometry was used alone or in combination with other lymphedema assessment tools, it proved helpful in characterizing the lymphedema state in its various stages and judging the efficacy of various treatment modalities. The choice of which type of device to use in a given setting mainly depends on the specific nature of the intended measurement purpose. The present review provides relevant information to help make such choices.

## Introduction and background

Limb lymphedema occurs when the drainage capacity of the limb's lymphatic system is less than needed to remove fluid filtered from the capillary network [1-3]. This can occur due to reduced functional properties of the lymphatic network or increased resistance to lymph flow within the lymphatic network [4-6]. A well-known condition that may lead to lymphedema of the upper extremities is the treatment of breast cancer, often stated as breast cancer treatment-related lymphedema (BCRL) [7-9]. Similarly, lower extremity lymphedema may occur with the surgical and radiation treatment of gynecological [10-12], prostate cancers, or other conditions including filariasis [13]. Lymphedematous conditions are most often associated with limb swelling, which may be assessed by measuring changes in limb volumes [14-16], and has been shown to most often progressively increase over time if untreated [17-19]. Moreover, lymphedema frequently causes temporal changes in the affected limb's skin and underlying tissue, likely affecting the tissue’s mechanical properties. Reported changes include epidermal thickening and increases in dermal collagen [20], dermal thickening [21], thickening of the hypodermis [22], and fibrosis [23-25]. Such changes and fluid increase are likely to decrease the lymphedematous limb's compressibility, perhaps in proportion to the extent of lymphedema-related changes in tissue mechanical properties and excess fluid volume. Consequently, measurements of changes in the skin's compressibility may help evaluate the magnitude of progressive increases in lymphedema or, contrastingly, evaluate non-volume-related treatment improvements. One noninvasive and easily measurable parameter related to the skin’s compressibility is its indentation resistance, which has been defined as the ratio of the tissue indentation force (TIF) to the tissue indentation distance (TID) [26]. A measurement device that applies a fixed force and determines the resulting indentation distance has been referred to as a tonometer, and, in what follows, will be referred to as type A. An alternate approach is to indent to a certain distance and measure the force required; this will be referred to as type B. Indentation devices operating on each principle have been used [27,28]. From a mathematical, conceptual view, the relationship between the elastic indentation force (F) and displacement (D) of tissue with an overall thickness of H by a cylindrical-shaped indentor may be expressed as F = D x E x W x ((K / (1 - p^2^)). In this equation, E is the tissue’s elastic modulus, W is the indentor width, p is the tissue’s Poisson ratio, and the factor K depends on the ratio of D/H and W/H [29-31]. However, when using a given indentor device on a given tissue, W, K, and p are relatively constant, and thus the ratio F/D mainly depends on the tissue’s elastic modulus. For a given tissue, the indentation force depends on the penetration depth [32]. The indentation resistance (F/D) is expected to increase as the tissue becomes more fibrotic and is expected to decrease with effective treatment. This narrative review aims to describe the various indentation methods and their applications and report on the clinical findings and utility of such measurements when used in patients with limb lymphedema of any cause.

## Review

Methods

Search Criteria and Process

Three databases (Web of Science, PubMed, and Embase) were searched for peer-reviewed, original study articles written in English on skin indention or tonometry in limb lymphedema from 1970 through 1/12/2025 that included indentation data.

Search Method

**Figure 1:**
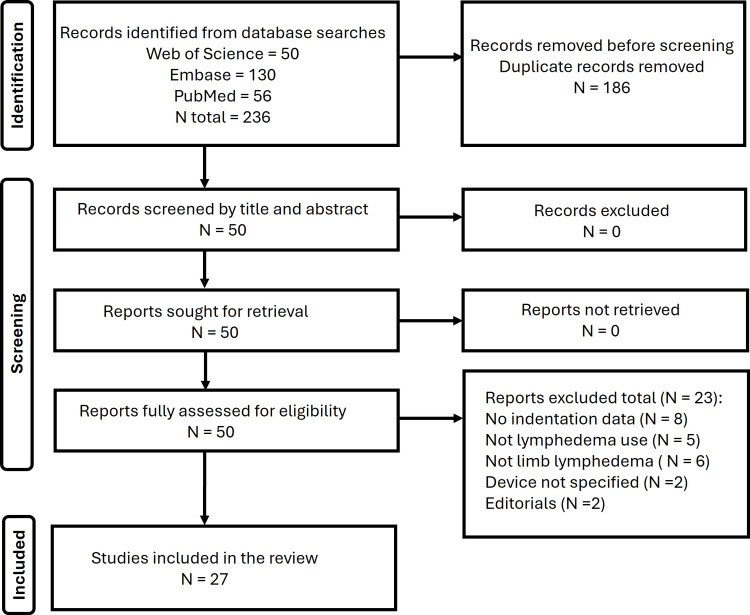
Search process

For the Web of Science search, the Boolean search string was as follows: ((ALL=(indent*)) OR ALL=(tonom*)) AND ALL=(lymphedema). This yielded 50 peer-reviewed articles written in English. A corresponding search string was used in EMBASE, which yielded 130 articles, and PubMed, which yielded 56 articles for a total of 236 records, as shown in Figure 1.

These 236 records were examined for duplicates, and 186 were found and removed, leaving 50 articles retrieved in full and reviewed. Of the 50 articles, eight were excluded because the article had no or inadequate indentor data, five were excluded because lymphedema was not a target of the article, six were excluded because measurements were not done on a limb, two were excluded because the device was not specified, and two were excluded as editorials. The total number excluded for these purposes was 23, leaving 27 for inclusion in the review section of this report.

Result summary

Table 1 summarizes the significant elements of the 27 studies that will be considered in further detail in the Discussion section. In 18 of these studies, some form of lymphedema treatment was evaluated. In these studies, type “A” devices were used in 11 studies and type “B” was used in seven studies. In nine studies, specific aspects of the lymphedematous condition were evaluated, but no treatment was evaluated. In these studies, type “A” devices were used in six studies and type “B” in three studies.

**Table 1:** Summary of results. Type “A” indentor uses a fixed TIF and measures TID. Type “B” indentor uses a fixed TID and measures TIF. NA, not applicable; NG, not given in the publication; none, no treatment given; TID, tissue indentation distance; TIF, tissue indentation force; variable, wide range with values not always provided

Author and Year	Lymphedema Subjects Studied (N)	Control Subjects Studied (N)	Anatomical Sites Measured	Device Type	Applied or Developed Force (TIF)	Indentation Distance (TID, mm)	Indentor Base Plate Diameter (mm)	Indentor Width (mm)	Treatment	Outcome Comments
Clodius et al., 1976 [33]	12	12	Forearm	A	60-120-180 g	1-6	50	10	None	TID statistically less for long-term lymphedema
Piller et al., 1976 [34]	18	16	Forearm	A	NG	NG	50	10	Drug intervention	TID increased with increased duration of Venalot® treatment
Chen et al., 1988 [35]	17	0	Forearm	A	30 g	NG	NG	10	Lymphovenous bypass	TID increased after lymphovenous bypass in most patients
Piller et al., 1988 [ 36]	40	0	Affected and healthy arm or leg	A	NG	NG	50	10	Benzopyrone intervention	TID increased in a cross-over design with treatment
Kar et al., 1992 [37]	63	26	Posterior calf	A	70-140-210 g	2-3	50	10	None	TID is greater in patients with filarial lymphedema
Liu and Olszewsk, 1992 [38]	9	5	Posterior calf	A	NG	NG	50	10	Microwave heating	TID differential between legs is reduced after treatment
Piller and Thelander, 1998 [39]	10	0	Forearm	A	NG	NG	50	10	Low-level laser therapy	TID differential between arms is reduced during treatment but regressed after treatment
Szuba et al., 2002 [40]	27	0	Forearm	A	60 g	1.9 – 2.2	50	10	Intermittent pneumatic compression	No clear improvement in TID with treatment, although some limb volume reduction
Carati et al., 2003 [41]	33	28	Upper arm and forearm	A	216 g	NG	50	10	Low-level laser therapy	TID decreased initially, but follow-up suggested improvement but only in the upper arm
Mayrovitz et al., 2005 [42]	15	0	Forearm	A	360 g	NA	NA	15	Manual lymphatic drainage	Indentation recovery time less compared to pre-treatment (p<0.01)
Chen et al., 2008 [43]	17	0	Forearm	A	200 g	NG	60	10	None	Improved device evaluated for repeatability
Mayrovitz, 2009 [44]	11	12	Legs	B	200-400 g	4.0	30	10	Manual lymphatic drainage	TIF was reduced compared to pre-treatment (p<0.001)
Gordon et al., 2011 [45]	25	28	Legs	A	200 g	3.7 – 5.6	60	10	None	TID in the posterior thigh was greater in patients testing positive for lymphatic filariasis (p<0.01)
Mayrovitz and Davie, 2011 [46]	72	0	Legs and arms	B	200-400 g	3 – 4	30	10	Low-level laser therapy	TIF was reduced compared to pre-treatment (p<0.001)
Louden et al., 2014 [47]	12	11	Arms	A	200 g	NG	60	10	Yoga classes for eight weeks	TID does not appear to be altered from the baseline between groups at eight weeks
Mayrovitz and Yzer, 2017 [48]	20	0	Arms	B	204-408 g	4.0	30	10	Cooling of the arm	TIF was reduced compared to pre-treatment (p<0.001).
Sun et al., 2017 [49]	45	15	Legs	B	5-20 g	1.3	22	2.2	None	TID force correlated with the stage of lymphedema and collagen content
Hara and Mihara, 2018 [50]	6	0	Medial thigh	A	51 and 204 g	NG	NG	NG	None	TID correlated with the stage of lymphedema (r = 0.79)
Douglass et al., 2019 [51]	50	46	Legs	A	200 g	NG	70	10	Preventative chemo	TID differences present at pre-treatment were mitigated with treatment
Zaleska and Olszewski, 2019 [52]	52	0	Legs	B	Variable	1.3 and 10	22	11.3	Intermittent pneumatic compression	TID force reduced after 60 minutes of intermittent pneumatic compression treatment
Douglass et al., 2020 [53]	71	71	Legs	A	200 g	NG	70	10	Enhanced vs. standard care	Greater improvement in tissue compressibility in those patients receiving enhanced care.
Sano et al., 2020 [54]	25	0	Legs	A	400 g	0-3	NG	NG	None	TID tends to decrease with increasing lymphedema stage
Yu et al., 2020 [55]	90	0	Legs 56 Arms 34	B	8.5 – 16.3 g	1.3	22	2.2	None	TIF is larger in limbs with lymphedema and correlates with the lymphedema stage
Zaleska and Olszewski, 2020 [56]	52	0	Legs	B	Variable	1.3 and 10	22	11.3	Manual roller compression	There is no effect at a TID of 1.3 mm, but reduced TIF at 10 mm indentation
Mehrara et al., 2021 [57]	9	0	Arms	B	Variable	0.3	22	2.2	Experimental monoclonal drug	Reduced TIF after four months of treatment
Mayrovitz et al., 2023 [58]	30	0	Arms	B	22.1 -23.4 g	1.3	22	2.2	None	TIF is similar on medial and lateral standardized sites
Zaleska and Krzesniak, 2024 [59]	21	0	Legs	B	Variable	1.3 and 10	22	11.3	Short-term high compression	Three daily 30-minute high-compression treatments significantly reduced TIF and limb size

Discussion

Early Work

In 1976, Clodius et al. reported on applying a first-generation tonometer design, in which the device could be placed on the forearm, and the vertical displacement of a central indentor rod into the tissue could be measured using weights [33]. The patients in this study had long-standing lymphedema that averaged 8.3 years and had clinically determined fibrosis. The indentor displacement increased for both control and lymphedematous tissue when the tissue was loaded progressively with 60, 120, and 180 grams. However, at all loadings, the lymphedematous patients had significantly less indentation (p < 0.01). Another finding was that the indentation resistance was no different than in the controls in an additional small group of patients (N =4) who had lymphedema but had been treated with the drug Venalot®, a combination of the benzopyrones coumarin and troxerutin. This drug intervention component was among the first studies to use indentation measures to assess potential treatment outcomes, albeit in a tiny experimental group. An extension of this finding was reported in the same year, during which the drug Venalot was evaluated in 18 patients using the same tonometer device, and the effectiveness was compared to 16 lymphedematous patients not receiving the drug [34]. Two important findings emerged from this study. One indicated that the indentation resistance increased with increasing excess limb volume. Expressed in terms of TID, this was reported at around 1-mm displacement increase for each centimeter increase in arm circumference. However, it should be noted that the TID is related to the width (surface area) of the particular indentor. Smaller surface areas penetrate further for the same TIF. The second finding was that the indentation resistance decreased depending on the duration of Venalot usage. This outcome was attributed to its lysing action on proteins, which theoretically might remove the trigger for further collagen deposition and some existing fibrous material. Twelve years later, in 1988, Chen et al. used a different indentor device to assess the impact of lymphovenous bypass on 17 patients with arm lymphedema [35]. They reported their indentation results for up to nine months after the surgical procedure using the difference between the lymphedematous and the non-affected contralateral arm. Unfortunately, complete displacement values were not provided. Nonetheless, they reported that the patient's post-operative judgment of the arm softness was better correlated with the indentor-measured change than the arm volume change measured by water displacement methods. In the same year, Piller et al. used the original first-generation tonometer to assess the effects of benzo-pyrone treatment on patients with arm or leg lymphedema [36].

Field Measurements

**Figure 2:**
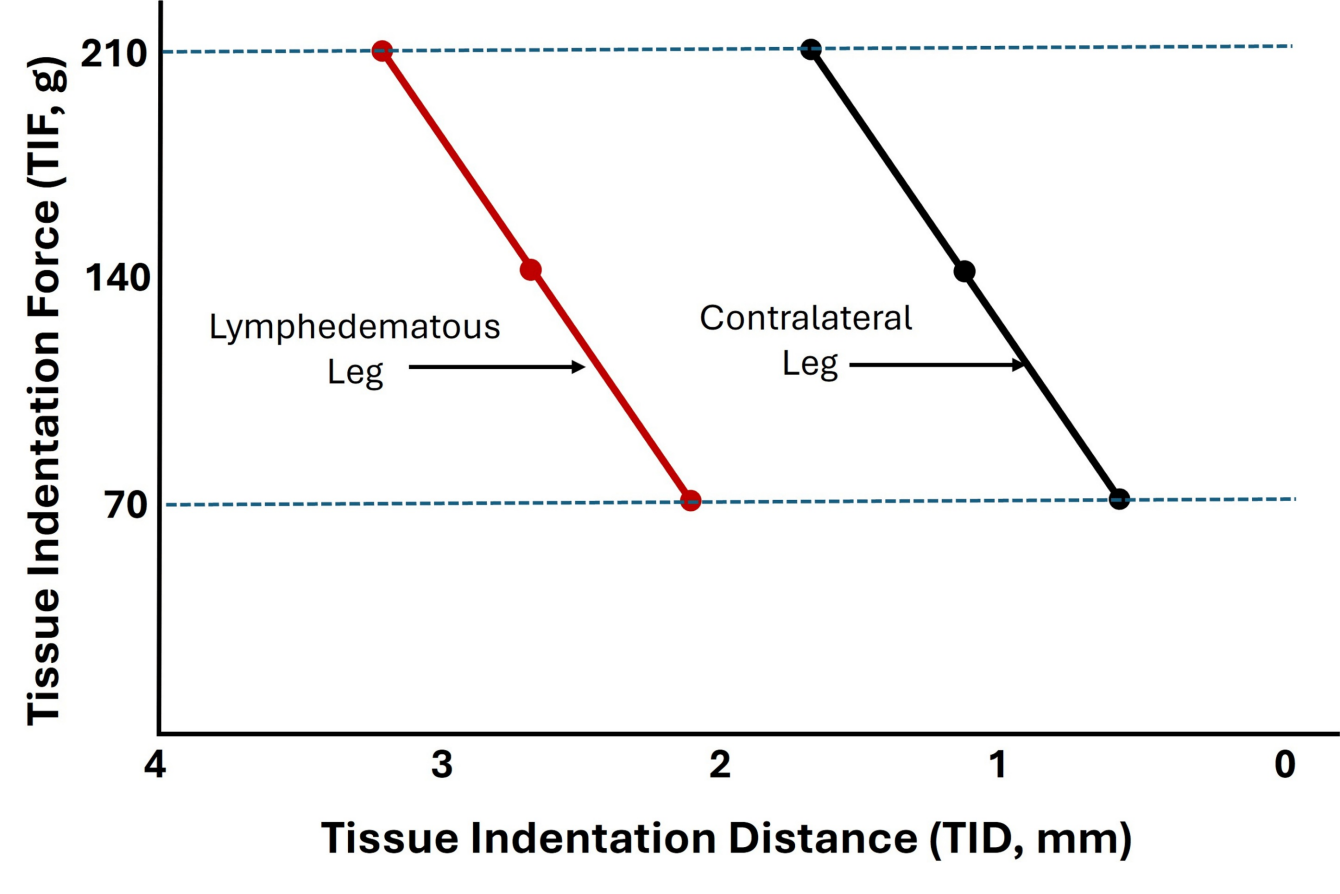
Illustration of leg lymphedema effect on indentation distance Patients had filarial-related unilateral leg lymphedema. The lymphedematous leg indentation distance was reduced at every indentation force compared to the contralateral leg. The figure is courtesy of Dr. H.N. Mayrovitz and is based on approximate data from Kar et al. [37].

Subsequent work was conducted using a modified device suitable for measurements in the field and applied to assess patients with filarial-related lower extremity lymphedema [37]. In this study, 63 patients were evaluated using TIF values of 70, 140, and 210 g on the posterior calf of the affected and contralateral non-affected legs while in a prone position. Figure 2 was created by the present author based on the average data reported in their paper. The results demonstrate the reduced TID of the lymphedematous leg compared to the other leg. This initial finding is characteristic of the limb lymphedema condition and varies with the stage of the lymphedema.

In the same year (1992) and using the same tonometer design, another group evaluated the effect of microwave treatment on nine patients with lower extremity lymphedema [38]. They reported that the differential in TID values between the lymphedematous and the non-affected contralateral leg was reduced after microwave treatment. This treatment consisted of heating to a maximum of 40oC during a 45-minute session for 15 consecutive days and was repeated twice more after a seven-day interruption. The authors concluded that this represented a normalization in tissue tonicity.

Assessing Treatment-Related Effects

The merits of treating BCRL with low-level laser therapy (LLLT) were assessed in 10 patients with several quantitative measures, including tonometry on the upper arm and forearm of the affected and contralateral arms [39]. The reported results were inconclusive since the forearm TID was stated to increase during treatment but reverted once treatment stopped. Furthermore, in this study, the upper arm showed a progressive decrease in TID, indicating tissue hardening.

The efficacy of intermittent pneumatic compression (IPC) added to standard therapy for BCRL was another intervention for which indentation measures were utilized. In one study, the impacts of IPC were assessed using several parameters, including indentation resistance in 27 patients [40]. Although the IPC addition was reported to decrease limb volume, changes in TID did not indicate a clear improvement in indentation resistance. Another study aimed at evaluating the impact of LLLT on BCRL with quantitative assessments, including tonometry [41]. Results indicated an initial hardening of the tissue (upper arm and forearm) with treatment. Still, with further treatment and follow-up, there was an indication of improved indentation resistance only in the upper arm. Overall, the outcome of the tonometry measurements in this study would be classified as indeterminate.

**Figure 3:**
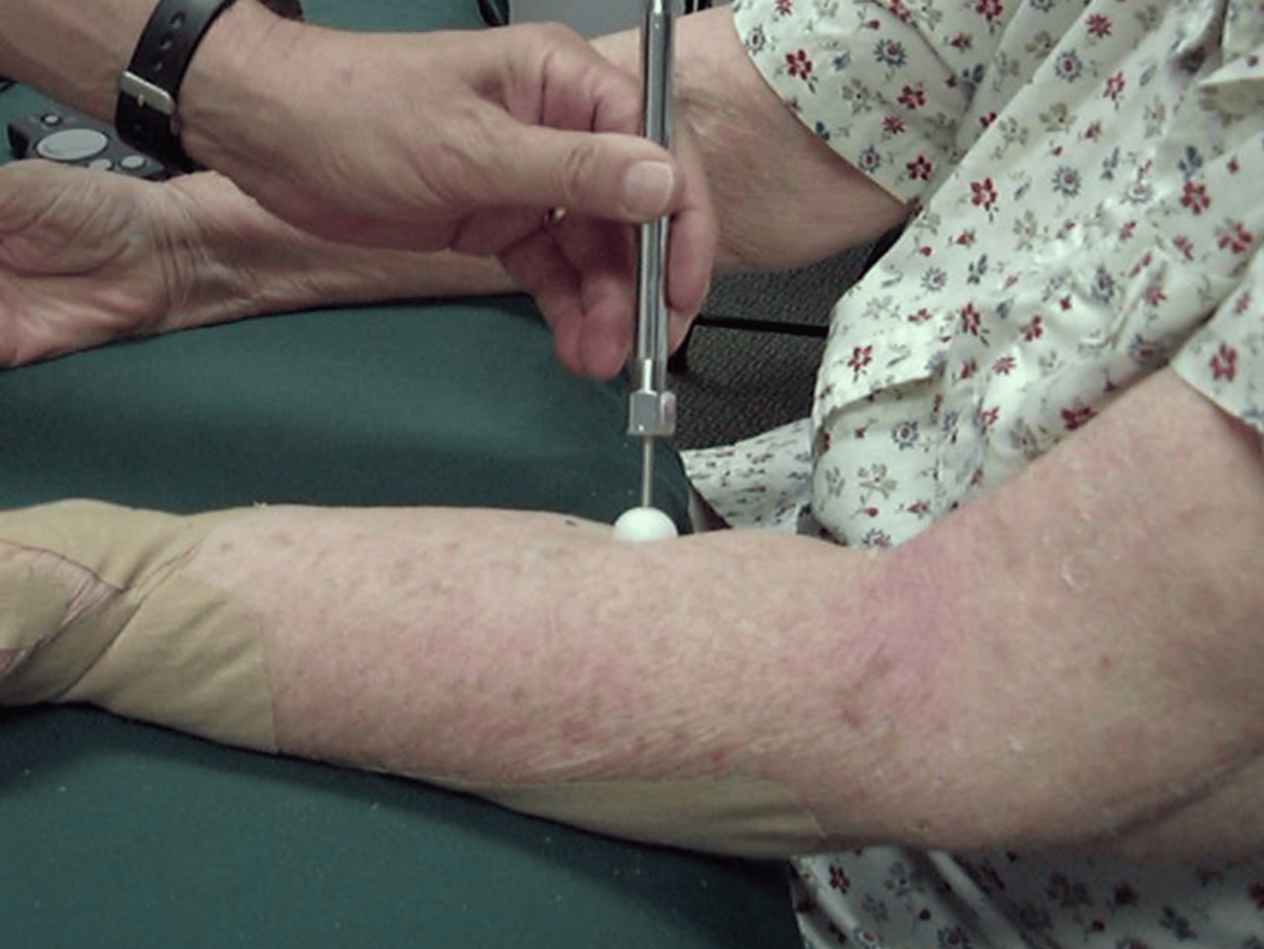
Illustration of indentation recovery time assessment A spherical indentor depresses the tissue at a fixed force for a fixed time, and the indentation's recovery time is determined visually and by palpation. The figure is a courtesy of Dr. H.N. Mayrovitz.

A slightly different indentor format was used as part of a study to evaluate the effects of manual lymphatic drainage (MLD) on 15 patients with unilateral BCRL [42]. Forearms were indented with a 15-mm diameter spherical tipped indentor at a fixed force of 360 g, as illustrated in Figure 3. Then, the recovery time of the tissue indentation was determined before and after an MLD treatment. Results indicate a significant decrease in recovery time (p < 0.01), although the recovery time remained more than measured in the contralateral unaffected arm. This method could be used with a fixed indentation distance and different spherical dimensions.

**Figure 4:**
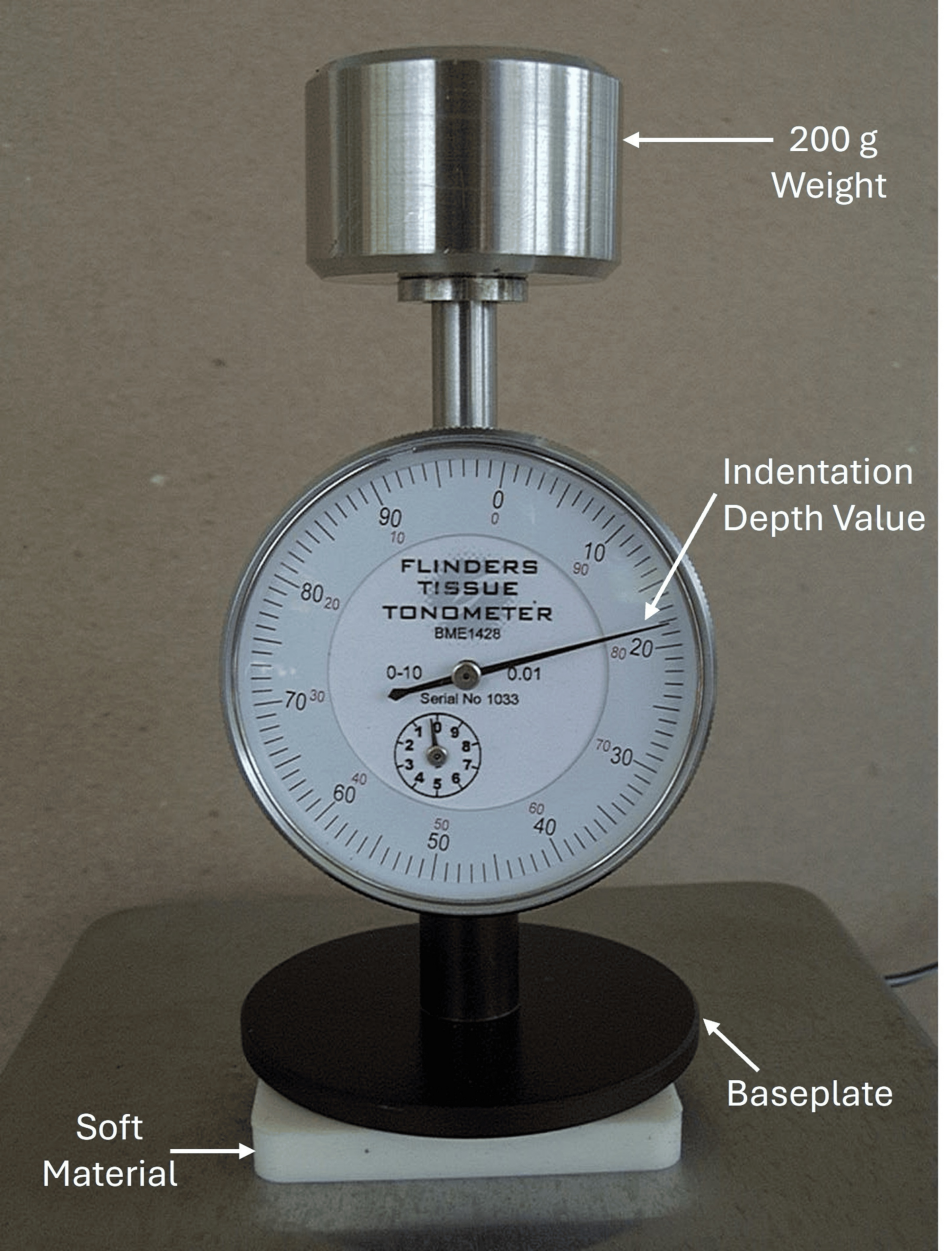
Illustration of a commercial indentation device The central 10-mm diameter indentor (not visible) indents into the material a distance recorded by the gauge dial. In this figure, the baseplate, which is 60 mm in diameter, rests on a soft material for illustration. When placed on the anterior forearm, issues of flatness and stability must be dealt with. The device with the weight removed weighs about 190 g. The figure is a courtesy of Dr. H.N. Mayrovitz.

In 2008, Chen et al. evaluated the reliability of an improved indentor device that had become commercially available [43]. This device, illustrated in Figure 4, was used in 17 patients who had developed BCRL. On the forearm, its intrarater intraclass correlation coefficient (ICC) was 0.879, and its interrater ICC was 0.714. This device was later used in several other studies which will be discussed later.

**Figure 5:**
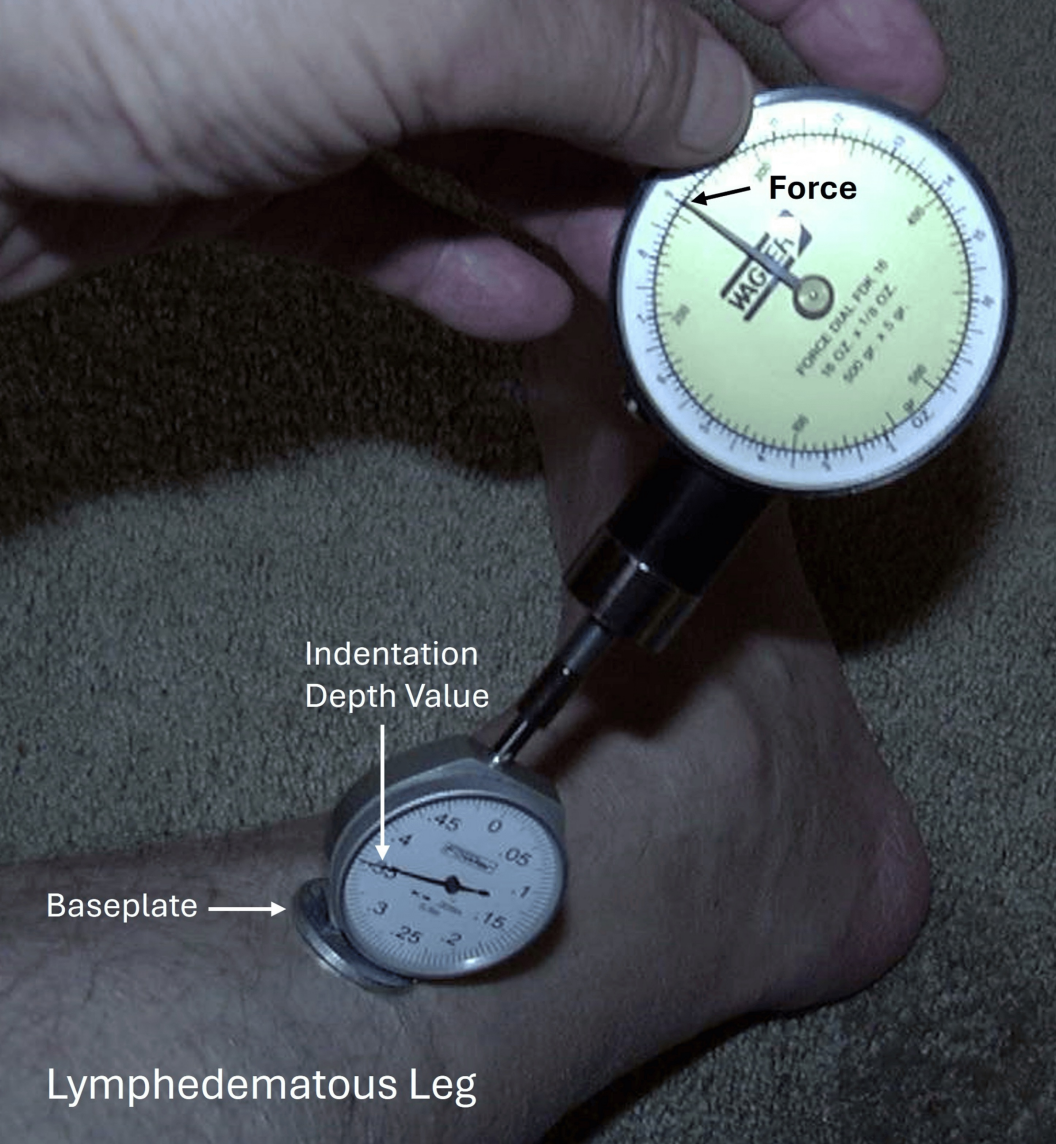
Illustration of a type "B" mechanical indentor A type “B” indentor is one in which the TID is fixed and the TIF is measured. In this instance, the indentor is pushed toward the leg until the desired indentation depth is achieved. The required force to achieve this (TIF) is measured. The indentation resistance can be determined as TIF/TID. The figure is a courtesy of Dr. H.N. Mayrovitz. TID, tissue indentation distance; TIF, tissue indentation force

Because the device shown in Figure 4 depends on gravity, it needs to be used only in a vertical position, which can be a limitation. To circumvent this, an indentor device that could be used in any orientation was developed and evaluated in vitro on polyurethane foams and also in 11 patients (18 legs) with lower extremity lymphedema before and after treatments and 12 controls (24 legs) [44]. This device, illustrated in Figure 5, indented the tissue to a desired amount with the force required registered on the gauge. In this study, an indentation distance of 4 mm was chosen, and a single MLD treatment session was reported to significantly reduce this force from 401±123 g to 332 ± 98 g (p < 0.001).

The possibility of using tissue indentation as a marker for early detection of lower extremity lymphedema associated with lymphatic filarial was investigated by Gordon et al. using the device pictured in Figure 4 [45]. They measured anterior and posterior locations in 25 adolescent patients with lymphatic filarial and 28 who were not infected. Based on their measurements, they concluded that despite no differences in leg volume or bioimpedance between the two groups, the indentor-based measurement in the posterior thigh was significantly less (p < 0.01). This suggested to the original authors that indentor measurements might help estimate the likelihood of progression to lymphedema in these patients.

In 2011, another study aimed at evaluating the therapeutic effects of low-level laser treatment was reported [46]. In this study, 38 patients with lower extremity lymphedema and 38 patients with BCRL were evaluated using the indentor previously shown in Figure 5. In addition, parallel measurements of changes in skin water were assessed. It was reported that TIF measured in arms and legs after laser treatment was significantly less than pre-treatment (p<0.001). The average TIF for arms was reduced from 297 to 222 g, and for legs, it was reduced from 381 to 314 g. In both arms and legs, this reduction in indentation resistance was accompanied by a significant decrease in tissue water. A few years later in 2014, Louden et al. used the device shown in Figure 4 to evaluate the possible effects of an eight-week program of yoga in patients with BCRL [47]. Their data and analytic technique, using tonometry measures on the forearm and upper arm of the affected and contralateral arms, does not support a significant change attributable to the yoga program.

In 2017, the effect of cooling a lymphedematous arm in 20 women with long-standing unilateral BCRL was evaluated using indentation measures with the device previously shown in Figure 5 [48]. This study observed significant reductions in indentation resistance after cooling when the forearm tissue was indented to a distance of four mm. They reported that with the standardized TID of 4 mm, an average pre-cooling TIF of 400 g was reduced to 306 g (p < 0.001). However, this reduced value was still higher than the value of 204 g measured on the unaffected contralateral arm. Interestingly, this change occurred despite no change in skin water content due to the cooling. This suggests that the effect was due to a cooling-related shift in the tissue component softness rather than a reduction in excess edema.

**Figure 6:**
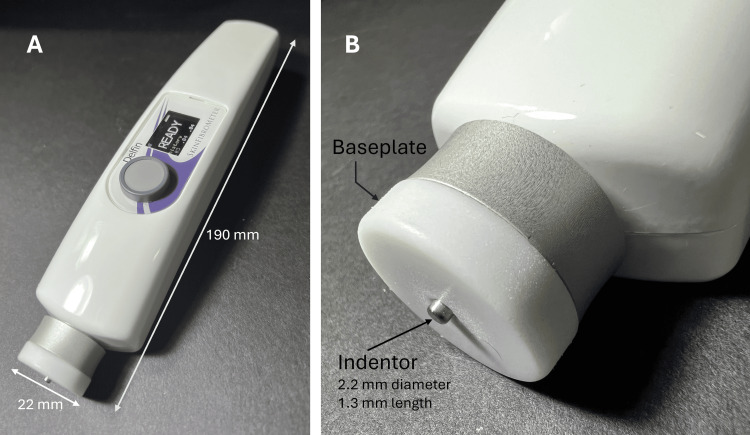
Illustration of an electronic type “B” indentor A type “B” indentor is one in which the TID is fixed and the TIF is measured. The completed device is shown in part A, and a close-up of the indentor and baseplate are shown in part B of the figure. This device's indentor is gently pushed against the skin multiple times. The required force to indent 1.3 mm for each push is recorded and averaged with the value indicated on the display. The figure is a courtesy of Dr. H.N. Mayrovitz. TID, tissue indentation distance; TIF, tissue indentation force

In the same year, Sun et al. employed an entirely different type of commercial indentor device, shown in Figure 6, to evaluate fibrosis in the skin of 45 patients with lower extremity lymphedema [49]. This device's indentor is gently pushed against the skin multiple times. The required force to indent 1.3 mm for each push is recorded and averaged with the value displayed in Newtons. This study reports that the TIF showed a strong positive correlation with the stage of lymphedema (r = 0.9, p < 0.01). The reported average forces for stages I, II, and III (converted from Newtons to g-force) were 7.25 g, 11.10 g, and 16.4 g compared to control limbs, with an average of 6.73 g. These increases were consistent with histologically determined increases in skin collagen.

A different indentor device was used to assess six patients with bilateral lower extremity lymphedema as a result of gynecological cancer treatment [50]. Few details of the indentor design were given. Still, when forces of approximately 51 g and 204 g were used on the medial thigh region, the indentation distance was also reported to correlate with the stage of the lymphedema.

Electronic Devices Emerge

In 2019, an electronic version of the mechanical device shown in Figure 3, referred to as an indurometer, was used to determine if young persons (10-21 years) with a diagnosis of lymphatic filariasis (LF) but asymptomatic might benefit from prophylactic preventative chemotherapy [51]. Based on the lower extremity indentor data obtained on 50 LF cases and 46 controls, baseline pre-treatment differences in TID at a fixed load of 200 g between LF-positive and LF-negative groups were effectively mitigated after preventative chemotherapy.

In another study focused on the utility of IPC, an extensive assessment of tissue properties of 52 patients with lower extremity lymphedema was undertaken using multiple measurement devices by Zaleska and Olszewski [52]. Among these measurements, they employed an indentor device similar to the one shown in Figure 5 to measure “deep” properties and the device shown in Figure 6 to evaluate shallower tissue properties. The deep properties were determined by indenting to 10 mm with an indentor diameter of 11.3 mm and the shallower to a depth of 1.3 mm with and indentor with a diameter of 2 mm. The force developed assessed the indentation resistance in each measurement set. They determined that the 45-60-minute IPC treatment reduced the resistance only in the calf region, with other leg areas remaining insignificantly changed. In the calf region, the pre-treatment average force was reduced by IPC treatment from 40.8 g to 18.4 g at the 1.3-mm indentation distance. This illustrates the utility of localized indentation measurements when anatomical variations in outcomes are present.

Further studies on patients with LF-related lower extremity lymphedema were conducted in 2020 by Douglas et al. [53]. An electronic version of the device, as shown in Figure 3, and other measures were used to assess the impact of standard versus enhanced care over a four-week interval in 142 patients, equally divided by care group. The results of their study showed that the indentation measurements were able to detect increased improvement in tissue compressibility in those patients receiving enhanced care. This finding applied to all lymphedema stages (p < 0.001) for a clinically relevant threshold of 10% improvement.

A different indentor design was employed by Sano et al. to evaluate the lower extremity tissue properties of 25 lymphedema patients [54]. The device they used was a commercially available one that measured the TID of a small diameter pin. Their results indicated a trend for decreasing TID with increasing lymphedema stage when measurements were done on the thigh using a fixed TIF. Comparable findings were reported by Yu et al., who used a device similar to that shown in Figure 6 to evaluate 56 patients with lower extremity lymphedema and 34 patients with BCRL [55]. These patients mainly presented with stage II and III lymphedema (91%), with an average duration of their lymphedema of four years. Average TIF values for control limbs indented to 1.3 mm on arms and legs were 8.5 g and 9.2 g, respectively. Corresponding values for the affected limbs were significantly greater (p < 0.05), being 13.3 and 16.3 g, respectively. These increases in indentation resistance, expressed as skin stiffness by the authors, correlated with the limb lymphedema stage (r = 0.68).

An interesting approach to the treatment of lower extremity lymphedema using a hand-operated roller to compress tissue to 80-120 mmHg was reported by Zaleska and Olszewski [56]. This was done in 20 patients, and tissue properties were evaluated using the device shown in Figure 6. For TID of 1.3 mm, they reported no change in TIF following treatment. Contrastingly, using an indentation depth of 10 mm using a device similar to that shown in Figure 5, a significant reduction in indentation force occurred (p < 0.05). The authors concluded that this high-pressure compression modality mainly disperses underlying fluid. A device similar to that shown in Figure 6 but with an indentor length of only 0.3 mm was used by Mehrara et al. to assess the effects of an experimental monoclonal interleukin-neutralizing antibody in nine women with BCRL [57]. Results of this pilot trial reported that despite no change in limb volumes, there was a significant improvement in skin compressibility (p < 0.02).

The dependence on the measurement site when using indentation measures was initially investigated using the device shown in Figure 6 to evaluate TIF values at a standard distance from the medial malleolus on the medial and lateral aspects [58]. Values obtained in 30 patients with edematous legs were reported to not differ between medial and lateral sites, with average values ranging between 22.1g and 23.4 g. These findings add to the accumulating knowledge of the role of such measurements. One of the most recent studies in which tissue indentation resistance was used aimed to evaluate the effect of short-term high-pressure compression on 21 patients with stage III lower extremity lymphedema [59]. A 10-cm-wide bandage was applied to the calf area with an interface pressure ranging from 58 to 120 mmHg for 30 minutes for three consecutive days to evaluate this. This process resulted in a 69.6 % reduction in skin stiffness, as measured with the device shown in Figure 6, and a 58.9% reduction in stiffness, measured with an indentation distance of 10 mm. Reductions in tissue water and calf circumference accompanied the indentation resistance change. The authors concluded that this initial treatment approach was superior to others when treating stage III lymphedema.

## Conclusions

The present review examines the evolution of tissue indentometry as an adjunctive tool in assessing limb lymphedema over the past 38 years. Its uses range from evaluating naturally occurring changes in lymphedematous skin using shallow and deep indentations to using these measures to evaluate various lymphedema treatment modalities. In all cases investigated, whether indentometry was used alone or in combination with other lymphedema assessment tools, it proved useful in characterizing the lymphedema state in its various stages or judging the efficacy of multiple treatment modalities. The choice of which type of device to use in a given setting mainly depends on the specific nature of the intended measurement purpose. The present review provides relevant information to help make such choices.
